# Tumor-Promoted Changes in Pediatric Brain Histology Can Be Distinguished from Normal Parenchyma by Desorption Electrospray Ionization Mass Spectrometry Imaging

**DOI:** 10.3390/biomedicines12112593

**Published:** 2024-11-13

**Authors:** Ana L. Seidinger, Felipe L. T. Silva, Mayara F. Euzébio, Anna C. Krieger, João Meidanis, Junier M. Gutierrez, Thais M. S. Bezerra, Luciano Queiroz, Alex A. Rosini. Silva, Iva L. Hoffmann, Camila M. M. Daiggi, Helder Tedeschi, Marcos N. Eberlin, Livia S. Eberlin, José A. Yunes, Andreia M. Porcari, Izilda A. Cardinalli

**Affiliations:** 1 Boldrini Children’s Center, Campinas 13083-210, Brazil; felipeluztorres@outlook.com (F.L.T.S.); ma.euzebio@gmail.com (M.F.E.); meidanis@unicamp.br (J.M.); t243567@dac.unicamp.br (T.M.S.B.); gradanat@yahoo.com.br (L.Q.); iva@boldrini.org.br (I.L.H.); camis.maia@icloud.com (C.M.M.D.); htedeschi@hotmail.com (H.T.); andres@boldrini.org.br (J.A.Y.); izilda@boldrini.org.br (I.A.C.); 2 Graduate Program in Genetics and Molecular Biology, Institute of Biology, State University of Campinas, Campinas 13083-970, Brazil; 3 Department of Chemistry, The University of Texas at Austin, Austin, TX 78712, USA; akrieger@utexas.edu; 4 Institute of Computing, State University of Campinas, Campinas 13083-852, Brazil; 5 MS4Life Laboratory of Mass Spectrometry, Health Sciences Postgraduate Program, São Francisco University, Bragança Paulista 12916-900, Brazil; juniermarreroguti@gmail.com (J.M.G.); alexrosinisilva@hotmail.com (A.A.R.S.); andreia.porcari@usf.edu.br (A.M.P.); 6 Faculty of Medical Sciences, State University of Campinas, Campinas 13083-887, Brazil; 7 Department of Neurology, Division of Neurosurgery, State University of Campinas, Campinas 13083-888, Brazil; 8 MackMass Laboratory for Mass Spectrometry, School of Engineering, PPGEMN & Mackenzie Institute of Research in Graphene and Nanotechnologies, Mackenzie Presbyterian University, São Paulo 01302-907, Brazil; marcos.eberlin@mackenzie.br; 9 Department of Surgery, Baylor College of Medicine, Houston, TX 77030, USA; livia.eberlin@bcm.edu

**Keywords:** pediatric oncology, brain tumor, intraoperative tool, mass spectrometry imaging, DESI

## Abstract

**Background:** Central nervous system (CNS) tumors are the second most frequent type of neoplasm in childhood and adolescence, after leukemia. Despite the incorporation of molecular classification and improvement of protocols combining chemotherapy, surgery, and radiotherapy, CNS tumors are still the most lethal neoplasm in this age group. Mass spectrometry imaging (MSI) is a powerful tool to map the distribution of molecular species in tissue sections. Among MSI techniques, desorption electrospray ionization (DESI-MSI) has been demonstrated to enable reliable agreement with the pathological evaluation of different adult cancer types, along with an acceptable time scale for intraoperative use. **Methods:** In the present work, we aimed to investigate the chemical profile obtained by DESI-MSI as an intraoperative surgical management tool by profiling 162 pediatric brain biopsies and reporting the results according to the histopathology and molecular profile of the tumors. **Results:** The 2D chemical images obtained by DESI-MSI allowed us to distinguish tumor-transformed tissue from non-tumor tissue with an accuracy of 96.8% in the training set and 94.3% in the validation set after statistical modeling of our data using Lasso. In addition, high-grade and low-grade tumors also displayed a distinct chemical profile when analyzed by DESI-MSI. We also provided evidence that the chemical profile of brain tumors obtained by DESI-MSI correlates with methylation-based molecular classes and specific immunophenotypes found in brain biopsies. **Conclusions:** The results presented herein support the incorporation of DESI-MSI analysis as an intraoperative assistive tool in prospective clinical trials for pediatric brain tumors management in the near future.

## 1. Introduction

Among all childhood solid tumors, those originating from the central nervous system (CNS) are the most frequent. Despite advances in pediatric neoplasm treatment due to protocol development guided by molecular classification, brain tumors remain the leading cause of cancer mortality and morbidity in children [1].

Brain tumors may vary from benign to highly malignant entities and can emerge in a diversity of anatomical regions in both infra- and supratentorial compartments [2]. Surgical handling plays a central role in CNS tumor management and occurs by fine-needle biopsy for histological diagnosis, cytoreduction, or complete excision [3]. Intraoperative histopathological diagnosis improves the surgical procedure, assisting the surgeon in the assessment of margins of excision and evaluation of the adequacy of the submitted tissue for downstream diagnostic procedures. Additionally, intrasurgical analysis enables the detection of suspicious lesions uncovered by clinical or radiological imaging, thus reducing the need for a second invasive procedure [4,5]. The current gold-standard technique for intraoperative diagnosis is the frozen tissue sectioning followed by the hematoxylin and eosin (H&E) staining protocol, developed in 1905 [6]. Despite its wide use, robustness, and affordability, there are important limitations imposed by this technique, such as the formation of water crystals that cause distortion of the tissue’s architectural detail, harming a reliable diagnosis [5]. Folding, crushing, and overstretching artifacts, as well as cautery from the surgery procedure can also affect the tissue’s architecture significantly [5]. Aside from these technical aspects, there is also the requirement of a trained neuropathologist, a good clinical history, neuroimaging, and specialized staff to provide higher intraoperative diagnostic accuracy [7]. Due to these difficulties, the overall concordance between intraoperative frozen section consultation and final diagnosis for brain lesions falls between 67% and 97% [8,9,10,11,12,13]. Agreement is lower in low-grade gliomas (82.5%) than in high-grade gliomas (93.2%) [13], and in 5% of cases, misdiagnosis of tumor versus non-tumor conditions may happen [12]. Noteworthy, it may take up to four biopsy analyses to increase the accuracy in diagnostic yield from 67% to 89% [8].

Cancer cells acquire functional capabilities that are crucial for their ability to form malignant tumors as they make their way from healthy to neoplastic growth states. Amongst these changes, altered abundance and types of lipids play a role in each of the hallmarks of cancer [14,15]. For example, ceramides with long-chain fatty acids are reported to have pro-apoptotic properties, whilst very long-chain fatty acid-containing ceramides are anti-apoptotic [16]. Several studies found lipid alteration in cancer, e.g., lysophospholipids in ovarian cancer [17], glycerophospholipids in hepatocellular carcinoma [18], acylcarnitines and glycerophospholipids in prostate cancer [19], sphingolipids in glioma [20] and breast cancer [21]. Evidence also suggests that choline-containing lipids and phospholipids increase in abundance during the metastasis process [22]. Once lipid classes show significative differences between tumor and non-tumor samples, changes in the lipid profile have been exploited for biomarker development, with the mass spectrometry-based approach emerging as a valuable tool in the field.

Mass spectrometry (MS) has gained attention from clinical audiences, as it can provide quantification of multiple biomarkers at a time, as well as enable discovery of molecules that pinpoint to novel biological processes related to disease development [23,24]. MS techniques encompasses multiple instrumentation types according to the choice of the inlet method, ionization source, and mass analyzer. One specific subset of MS techniques is defined by the analysis of samples in their native state, aiming to speed up the assay and minimize or eliminate sample preparation [25]. This subset is called ambient ionization MS (AIMS) and was introduced in 2004, with the invention of the Desorption Electrospray Ionization Technique (DESI-MS) and DART [26,27]. There are a variety of related AIMS techniques being used for intrasurgical tissue analysis such as rapid evaporative ionization MS (REIMS) [28,29], SpiderMass [30,31], the MasSpec Pen [32,33,34], and the Picosecond Infrared Laser MS (PIRL-MS) [35,36].

DESI-MS offers the possibility of profiling the molecules present on the surface of the tissue as a function of their spatial distribution, thus generating an MS image that maps the molecule’s location without the need to use antibodies or markers. Although DESI has lower spatial resolution than other MSI techniques, it has advantages in the minimal need for sample preparation and open air analysis. DESI-MS imaging (DESI-MSI) of tissue sections is conducted under ambient conditions using nondestructive histologically compatible solvent systems that preserve the tissue architecture and allow subsequent analyses on tissue sections already screened by DESI, adding and correlating molecular and biological information retained from other techniques on the very same section [37]. The tissue section placed on a glass slide is mapped on the *x* and *y*-axis while it is sprayed with a charged solvent at high speed provided by a high-pressure flow of N_2_. The spray microdroplets extract biomolecules (mainly lipids and metabolites) from the tissue. The secondary microdroplets carrying the extracted molecules are further converted into gaseous ions through ESI-like mechanisms, allowing their detection and quantification in the mass spectrometer [38,39,40]. The molecular profile obtained for each *x-* and *y*-coordinates are converted into a 2D chemical image from the tissue. Ion intensities are displayed by using a false-color scale with the relative ion abundance reflected by the intensity of the color [41].

DESI-MSI has been successfully applied to identify different cancer types, such as liver [42], breast [43], kidney [44], prostate [45], bladder [46], gastric [47], pancreatic [48], thyroid [49], lung [50], and colorectal cancer tissues [51]. Different types of brain tumors have also been assessed using DESI-MS or DESI-MSI approaches. This research was led by Cooks et al. and evolved from demonstrating the ability of DESI-MSI to discriminate between types of brain tumor in adults, such as oligodendroglioma, astrocytoma, and oligoastrocytoma, up to the validation of DESI-MS for target detection of 2-hydroxyglutarate (2-HG) in a large human glioma cohort of adult subjects, for intraoperative isocitrate dehydrogenase (IDH) mutation assessment [52,53,54,55,56,57]. With a focus on pediatric patients, Woolman et al. (2024) recently applied PIRL-MS to differentiate between medulloblastoma and pilocytic astrocytoma, as well as two ependymoma molecular subtypes, using a set of 18 metabolic lipid markers [36]. Based on the molecular changes associated with the tumorigenesis processes and focusing on developing reliable surgical devices that can improve and refine the intraoperative consultation tools, we aimed at investigating the use of DESI-MSI, especially for intrasurgical margin evaluation, in a large pediatric brain tumor cohort. Our results show that DESI coupled to low-resolution mass spectrometry imaging allows for robust classification of pediatric brain biopsies specimens and may serve as an assistive tool for pathology diagnosis during the intraoperative analysis of frozen sections. Furthermore, the profile obtained by DESI-MSI in this pediatric cohort showed correlation with the methylation-based molecular classification of tumors and specific immunophenotypes, illustrating that the approach has the potential to be used to deliver real-time molecular information intraoperatively thusimproving the decision-making process in pediatric neuro-oncology.

## 2. Materials and Methods

### 2.1. Study Design

A retrospective/prospective study was carried out encompassing tissue samples from patients diagnosed with primary or metastatic CNS tumors in our institution, submitted to a neurosurgical procedure aiming at diagnostic and/or therapeutic purposes. Retrospective tissue samples were collected between the years 2000 and 2003 and the prospective ones were collected between the years 2019 and 2021. The samples were snap frozen and long-term (if retrospective) stored at −80 °C. The exclusion criteria to assign a sample ineligible for downstream analysis included the following: (i) samples presenting low signal intensity after ionization, (ii) low tissue adhesion to the glass slide, or (iii) very small sample size, preventing the accurate assessment of its histological characteristics for further annotation.

### 2.2. Patients and Clinical Data

A total of 162 samples were obtained from 132 patients. The age of patients at diagnosis ranged from 6.2 months to 21.4 years, with a mean of 7.3 years and a median of 6.8 years. The diagnoses of patients included in this study are detailed in Supplementary Table S1. The tumor biopsies were diagnosed according to the WHO 2016 classification [58] by two medical pathologists. Data regarding sex, age, clinical manifestations, images, staging and clinical evolution were collected by clinical oncologists from the patient’s medical records.

### 2.3. Sample Preparation

Tissue specimens were sectioned using a CM 1850 Leica cryostat (Leica^®^ Microsystems, Wetzlar, Germany) with the chamber at −20 °C. Sections measuring 14 µm thick were mounted onto silanized glass slides and stored at −80 °C until the DESI-MSI analysis.

### 2.4. DESI-MSI

MS measurements were performed by using a 2D Omni Spray DESI imaging platform (Prosolia Inc., Indianapolis, IN, USA) coupled to a linear ion trap mass spectrometer (LTQ XL, Thermo Fisher Scientific, Waltham, MA, USA). Lab-built sprayers were adapted to the commercial Omni Spray DESI imaging stages [59]. DESI-MSI was performed on mounted tissue slides using dimethylformamide-acetonitrile (1:1 *v*/*v*), in the negative-ion mode. Solvent flow rate was 1.1 µL/min and N_2_ pressure was 150 psi. Ions over the mass range *m/z* 180–1200 (which we will refer to as the lipid profile) were acquired in the profile mode, performed with the sum of 2 microscans and an injection time of 350 ms. The automatic gain control was deactivated.

The raw data obtained by the mass spectrometer were grouped and converted into a 2D image using the software FireFly version 2.1.05 (Prosolia, Inc.), with a bin size of 0.083 Da, generating 12,240 values of *m/z* for each coordinate scanned. The generated images were later visualized with the Biomap software version 3.8.0.4 (Novartis, Basel, Switzerland).

### 2.5. Histopathological Analyses and Spectra Annotation

The same tissue sections which underwent DESI-MSI were subjected to standard H&E staining and visualized under a light microscope by the medical pathologist (I. A. Cardinalli). The regions of interest (ROI) in each biopsy were labeled according to their histological characteristics as normal tissue, necrosis, inflammation, keratin, calcification, grade I, II, III or IV malignant tumors, metastases, benign tumors, and malignant mesenchymal neoplasia. The corresponding MS spectra from each ROI were extracted using MSiReader software version 1.0 (North Carolina State University) with a *m/z* tolerance of 5 ppm. Both raw data for each pixel and averaged *m/z* abundance in the ROI were exported.

### 2.6. Statistical Analysis

The averaged mass spectra were normalized to the total ion current (TIC), log-transformed (base 10), and auto-scaled (mean-centered and divided by the standard deviation of each variable) [60,61,62]. The unsupervised clustering analyses were based in *t*-test/ANOVA and were conducted by using the MetaboAnalyst 5.0 platform [63]. The clustering method and distance measure are indicated for each analysis in the respective figure captions. To identify differentially abundant ions between groups, a non-parametric *t*-test was employed, using an FDR (false discovery rate) adjusted *p*-value ≤ 0.05, and at least 2× fold change cutoff. Due to the high number of variables (*m/z* values) considered in the analysis (>1000), 40% of the variables presenting near constant values between the groups were filtered out through the interquantile range filter.

### 2.7. Data Modeling

Data obtained from each pixel was compiled into an .xlsx file and the *m/z* values that were not detected in more than 10% of the pixels were removed. The mass spectra were normalized by log-transformed median ion intensity. Median normalization adjusts the log intensity values based on the global median value [64]. Data were grouped according to the nominal value of the masses. The spreadsheets containing the *m/z* values per pixel were processed using the Lasso regression analysis method (Least Absolute Shrinkage and Selection Operator) [65]. Inverse probability weighting was used to minimize the imbalance between classes. The Lasso model performance was evaluated using 5-fold cross-validation on the training dataset, as well as assessing performance on a withheld validation dataset comprising samples not included in model generation.

### 2.8. Collision Induced Dissociation Tandem Mass Spectrometry (CID-MS/MS)

CID-MS/MS was performed following the same conditions as the DESI-MSI for the initial acquisition. The precursor ion selection mode was used, and sufficient fragmentation energy was applied to fragment the ion of interest keeping its intensity at ~30% of the base peak. The acquired spectra were integrated for at least 30 s, using the average spectrum obtained for the assignment. The resulting fragmentation profiles were compared to the information present in the Lipid Maps database [66]

### 2.9. Immunohistochemistry Staining

After deparaffinization and rehydration, 5 µm-thick histological sections of primary CNS tumors (n = 50) were mounted on silanized slides, treated with H_2_O_2_, and then subjected to heat-mediated antigen retrieval. The sections were incubated with the primary antibodies according to Supplementary Table S2. The visualization system used was the EnVision FLEX Mini Kit, High pH (Agilent, Santa Clara, CA, USA). Positive controls were used in all batches.

Vascular micro density was evaluated through the average number of micro vessels in selected areas of the tumor. Slides labeled with the anti-CD34 antibody were initially examined at a lower magnification (40× or 100×) to identify areas of higher vascular concentration (“hotspots”) i.e., areas with higher intensity of CD34 expression. Ten high power fields (400×) were examined. The micro vessel count was expressed as the average of all evaluated fields.

### 2.10. Methylation-Based Molecular Classification of Tumors

Genomic DNA was extracted from tumor samples using the GenElute™ Mammalian Genomic DNA Miniprep Kit (Merck, Rahway, NJ, USA) according to the manufacturer’s instructions and quantified using the Qubit dsDNA HS Assay Kit (Thermo Fisher Scientific). A total of 200 ng of genomic DNA were converted by sodium bisulfite treatment with the EZ DNA Methylation-Gold Kit (Zymo Research, Irvine, CA, USA) as per the manufacturer’s instructions. The samples were hybridized with the Infinium MethylationEPIC Kit array, according to the manufacturer’s specifications (Illumina, San Diego, CA, USA).

Molecular classification of tumors was performed using a molecular neuropathology platform (MNP), jointly developed by the University Hospital of Heidelberg, the German Center for Cancer Research (DKFZ), and the German Consortium for Translational Research in Cancer (DKTK) [67]; it has been used for molecular classification of CNS tumors in Germany since 2015. The platform is based on the random forest algorithm and performs a prediction based on comparison with data from a reference cohort containing more than 2800 tumors of neural origin of almost all known entities (currently, more than 80 tumor classes or subclasses are included). The main result is a classification-calibrated score that indicates the similarity of the sample of interest to one of the included CNS tumor classes. A calibrated score between 0 and 1 is generated for each class. All class prediction scores theoretically add up to 1. For a reliable prediction, a methylation class score must be above the cutoff of 0.84 [68].

## 3. Results

### 3.1. 2D Ion Images Obtained by DESI-MSI Consistently Agree with Histological Architecture of the Tissue

**Figure 1:**
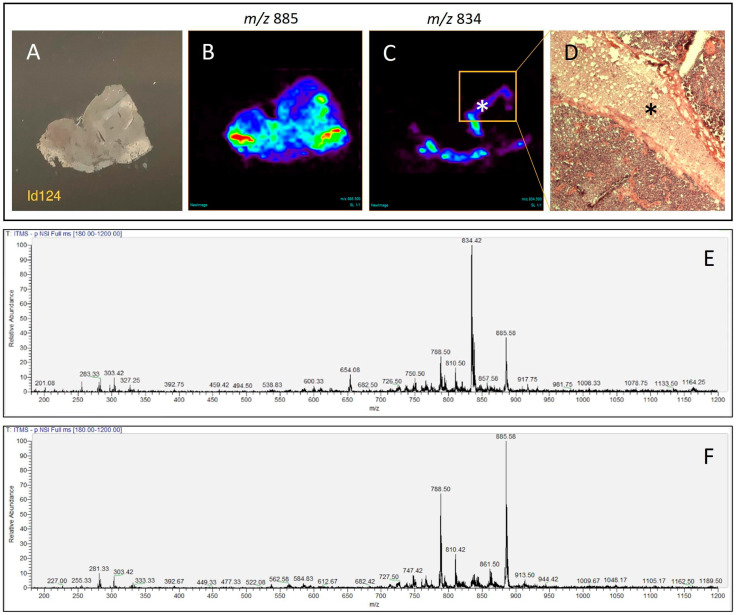
DESI-MSI analysis of a tissue section containing non-tumor and tumor tissue in the same biopsy. (**A**) Frozen section before DESI-MSI analysis. (**B**) Distribution of the ion *m/z* 885 across the tissue. (**C**) Uneven distribution of the ion *m/z* 834; the asterisk indicates the tissue region corresponding to the non-tumor brain parenchyma. (**D**) Histological examination of the section after analysis by DESI-MSI, stained with hematoxylin-eosin (40×). The asterisk corresponds to the same non-tumor brain parenchyma region seen in panel (**C**). (**E**) Mass spectrum corresponding to the non-tumor tissue region. (**F**) Mass spectrum corresponding to the tumor tissue region.

**Figure 2:**
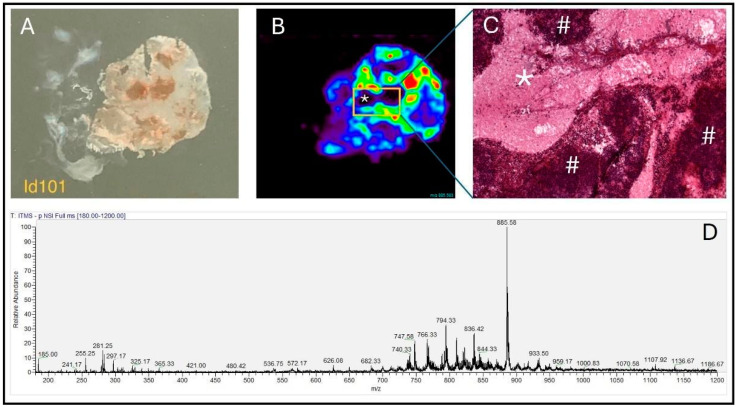
DESI-MSI analysis of a tissue section containing tumor tissue and necrosis in the same biopsy. (**A**) Frozen section before DESI-MSI analysis. (**B**) Uneven distribution of the ion *m/z* 885 across the tissue; the asterisk indicates the tissue region corresponding to necrosis. (**C**) Histological examination of the section stained with hematoxylin-eosin, after the analysis by DESI-MSI (40×). The photograph is equivalent to the region detailed by the yellow rectangle in panel (**B**). The asterisk corresponds to the same necrosis region seen in panel (**B**). The # symbol refers to regions of high cellularity, corresponding to tumoral cells. (**D**) Mass spectrum relative to the tumor tissue region.

The results obtained after analysis of the 162 pediatric brain tissue biopsies demonstrate that 2D chemical images generated by DESI-MSI agree with the histologic information observed, as illustrated in Figure 1, where normal and tumoral tissue findings were represented, and Figure 2, where analysis of a tissue section containing necrosis and tumoral tissue is depicted. The chemical image reproduces the architecture of the tissue analyzed, evidencing uneven distribution of ions throughout the sample, according to the tissue regions with different histological characteristics. In addition, the results of prospective (n = 76) and retrospective (n = 86) DESI-MSI analyses showed that no biases were identified due to the type of recruitment (prospective vs. retrospective samples), as shown in Supplementary Figure S1.

A subset of samples (n = 37; 23%) was excluded from the subsequent steps of this study due to the exclusion criteria (Supplementary Figure S2). The percentage of exclusion in the retrospective group was higher than in the prospective group, but not statistically significant (26% vs. 20%, respectively; χ2 test (1, N = 162) = 0.7, *p* = 0.37).

### 3.2. DESI-MSI Data Can Be Used to Distinguish Tumor Tissue from Normal Parenchyma in Pediatric Brain Biopsies

To investigate putative differences between the lipid profile from normal brain tissue and tumor brain tissue in the pediatric age group, 21 sets of mass spectra from normal brain parenchyma and 98 sets of mass spectra from tumor tissue were analyzed, regardless of diagnosis and/or staging.

**Figure 3:**
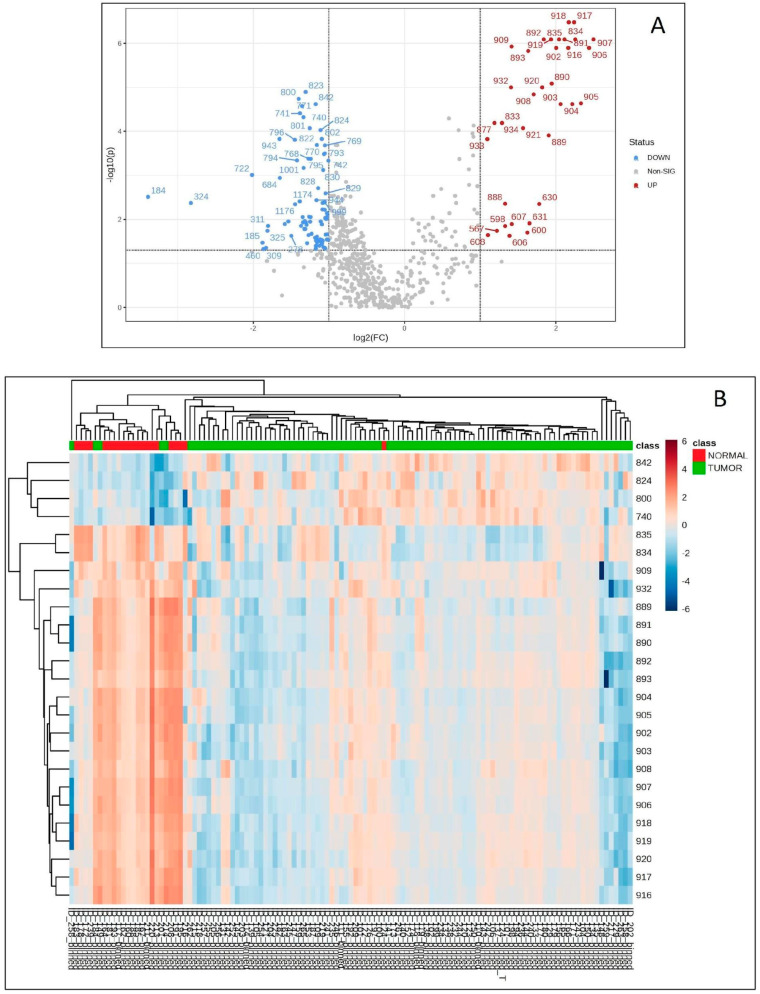
(**A**) Volcano plot depicting downregulated variables in the normal tissue in blue, upregulated variables in red, and non-significant variables in gray. Each dot represents one ion. (**B**) Hierarchical clustering analysis based on the average and Euclidean distance measure depicting the main 25 variables responsible for unsupervised grouping of samples according to their lipid profile into the normal parenchyma and tumoral tissue categories. The color bar represents the data values across the samples, where blue means lower ion abundance and red means higher ion abundance.

We identified 119 variables (ions) with a significantly different abundance between the two tissue classes analyzed (non-parametric *t*-test; FDR adjusted *p*-value: 0.05; Fold change > 2×) (Figure 3A). The list of the 119 differentially abundant ions found between normal and tumor tissues and the respective fold-change is presented in Supplementary Table S3. The hierarchical clustering analysis shows that the two categories of tissues present a different abundance of ions, and can be distinguished through their chemical profile, as illustrated in Figure 3B. There are only 5 of 98 tumors grouped with normal tissues, and 2 of 21 normal tissues grouped with tumors (Figure 3B).

To further explore if the differences found in the lipid profile of samples allowed distinguishing between normal and tumor-transformed brain parenchyma by using mass spectrometry data, we used the Lasso method [65] to build and validate a model (or classifier) that can predict the tissue type based on the DESI-MSI profile. The Lasso method can effectively select a model that includes only the most important predictors, making it simpler to interpret the selected metabolites’ contribution to the model [69]. This is particularly useful in high-dimensional datasets, such as the one produced by DESI-MSI, since the spectrum of each pixel produces a large number of ion signal features. The Lasso method has been employed in many DESI-MSI applications [40,43,48,70,71] and presents robustness to overfitting because the penalty term helps ensure that the model generalizes well to new data. Data modeling followed these general steps: (i) split data into training and validation sets; (ii) generate a model with the training data; and, (iii) insert test data in the model and compare the prediction to real labels. The whole dataset used for Lasso modeling comprised 36,408 pixels, from which 25,674 pixels labeled as non-normal (tumor-transformed) or normal were used to train the model, and 10,734 pixels were withheld from model generation and used to assess model performance. The Lasso method identified *m/z* values that are predictive of the two classes considering the lipid profile *m/z* > 700 from normal brain tissue (tissue with no histological evidence of tumor transformation) and those from the remaining eleven histological categories associated with tumor transformation (necrosis, inflammation, keratin, calcification, grade I, II, III or IV malignant tumors, metastasis, benign tumors or malignant mesenchymal neoplasia). By using this approach, the cross-validation analysis of all samples from the training set achieved an accuracy of 96.2%, and 94.3% in the validation set, compared with standard histopathologic evaluation (H&E) (AUC = 0.96) (Table 1). As a result of the combination approach DESI-MSI/Lasso modeling, we were able to identify molecules that are common to the tissue transformation process, allowing us to discriminate against the complexity of tumor-associated changes in brain histology when compared to normal brain parenchyma, regardless of tumor histology.

### 3.3. DESI-MSI Reveals Differences Between the Lipid Profile Found in Low-Grade Tumors Compared to High-Grade Tumors

**Figure 4:**
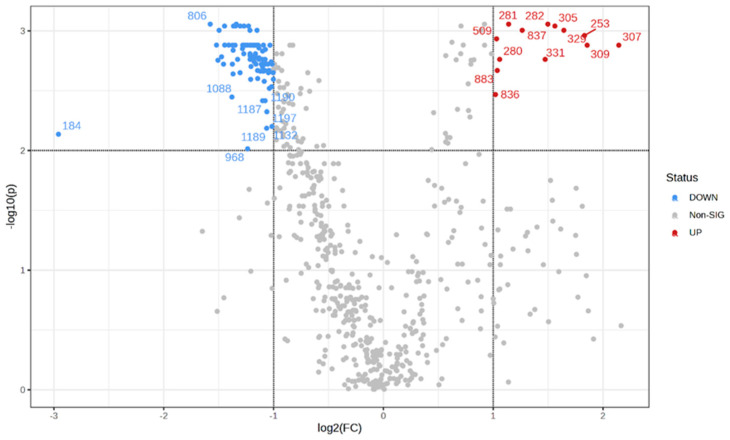
Volcano plot depicting the upregulated features in high-grade tumors in red, downregulated variables in blue, and non-significant variables between high- and low-grade tumors in gray. Each dot represents one ion and labels for the differentially abundant ions were provided.

CNS WHO grading relates to the clinical-biological behavior of tumors, which ultimately leads to differences in prognosis of patients. Treatment strategies, including surgical resection, are very different for low-grade and high-grade tumors [2]. Therefore, it is crucial to accurately assign tumor grade as soon as possible in the clinical management process. We investigated putative differences in the lipid profile between low-grade and high-grade tumors by analyzing 52 sets of mass spectra from low-grade tumors and 35 sets of mass spectra from high-grade tumors. For these analyses, only primary CNS tumors were considered. We identified 104 variables (ions) with significantly different abundances between the two groups and analyzed and built a volcano plot to depict the results (Figure 4) (Fold change > 2×; non-parametric *t*-test FDR-adjusted *p*-value 0.01). Our results showed that low-grade and high-grade tumors present a distinct lipid profile. The output list containing the 104 ions differentially abundant between the two groups (Supplementary Table S4) may serve as a basis for investigating the signaling pathways and mechanisms underlying acquisition of aggressiveness of brain tumors, especially in those cases in which a given tumor can evolve and be classified into different degrees of malignancy, such as astrocytoma, which can be classified as grade I (pilocytic astrocytoma), grade II (diffuse astrocytoma), grade III (anaplastic astrocytoma), or grade IV (glioblastoma).

### 3.4. Tandem Mass Spectrometry Allowed the Identification of Molecular Species Associated with Tumor Transformation in Pediatric Brain Tissue

The Lasso modelling of our data identified the ions of *m/z* 794, *m/z* 722, *m/z* 760, *m/z* 796, *m/z* 810, *m/z* 816, *m/z* 768, *m/z* 885, *m/z* 734, and *m/z* 772 as the top 10 features most predictive of tumor transformation in pediatric brain tissue, while the ions of *m/z* 834 and *m/z* 835 were predictive ofnormal brain parenchyma, according to their absolute value of the weight coefficient in the model (Table 1). In order to link specific molecular entities to the ions selected by the Lasso method, we conducted tandem mass spectrometry (MS/MS) utilizing collision-induced dissociation (CID) with a linear ion trap mass spectrometer for the five most relevant features of our model. CID involves the fragmentation of selected precursor ions in a reaction cell by colliding them with an inert gas. After the collision step, product ions are generated and then are subsequently analyzed by a second cycle of mass analysis, providing the fragmentation profile of the selected precursor ion based on its chemical structure. The resulting fragmentation profile is then queried against public databases, making it possible to identify ions of interest [72]. Although the LTQ-XL has nominal precision, our tentative ion identification allowed us to putatively identify the ion of *m/z* 794 as the chlorinated adduct of phosphatidylcholine (PC 34:1) and the ion most strongly associated with tumor transformation. The ion of *m/z* 834, [PS 40:6 –H]- was the most strongly associated with normal brain parenchyma (Table 2).

### 3.5. Mass Spectra Profile According to the Expression of Clinically Relevant Phenotype and Proliferation Biomarkers in Pediatric Brain Tumors

Immunohistochemistry (IHC) utilizes antibodies for the detection of specific antigens in tissue sections and it is widely used to refine the diagnosis of tumors because specific tumor antigens show marked alteration in cancer. It can help to define tumors of uncertain histogenesis and predict the prognosis of tumors by identification of biomarkers, such as enzymes, tumor-specific antigens, oncogenes, tumor suppressor genes, and tumor cell proliferation markers [73]. We interrogated the lipid profile of pediatric brain tumors according to the expression of clinically relevant phenotype and proliferation biomarkers determined by IHC in brain cancer. We found differentially abundant ions in pediatric CNS tumors immunopositive for CD34, BCL2, P53, B-catenin, EGFR, IGF1R, PDGFR-a, and VEGF when compared to immunonegative tumors (Nonparametric *t*-test, *p*-value ≤ 0.05). Although we have not found any differentially abundant ions when adjusting the *p*-value for FDR (q value 0.05), the unsupervised analysis showed that tumors immunopositive for CD34, BCL2, P53, EGFR, IGF1R, and VEGF clustered together based on their lipid profile when compared to tumors negative to the expression of these markers (Supplementary Figures S3–S8).

### 3.6. Mass Spectra Profile of Pediatric Brain Tumors According to Its Methylation-Based Classification

Genome-wide DNA methylation patterns have emerged as a promising tool to precisely define tumor classes and to improve diagnostic accuracy. The development of a CNS tumor classifier based on a DNA methylation array acts as a diagnostic adjunct in neuropathology, increasing diagnostic precision and reliable prognostic evaluation. For instance, for medulloblastomas and ependymomas, therapy decisions in University Hospitals of Heidelberg were often more heavily influenced by methylation class rather than morphological WHO diagnosis [74]. To understand how the lipid profile of brain tumors is related to their molecular classification, we obtained the global methylation profile for 128 tumor samples. Considering the cut-off of 0.84 [68], 97 samples (76%) were assigned into a known methylation class through the MNP classifier v.12.3 [67] and 81% of proposed classifications were in agreement with the histopathological diagnosis (n = 79).

To verify whether there is a correlation between the lipid profile of pediatric brain tumors and their methylation-based molecular classification, the samples were grouped according to the methylation class to which they belong, according to Table 3. Only groups containing more than three samples per group were included, totalizing 46 sets of chemical spectra of tumors.

**Figure 5:**
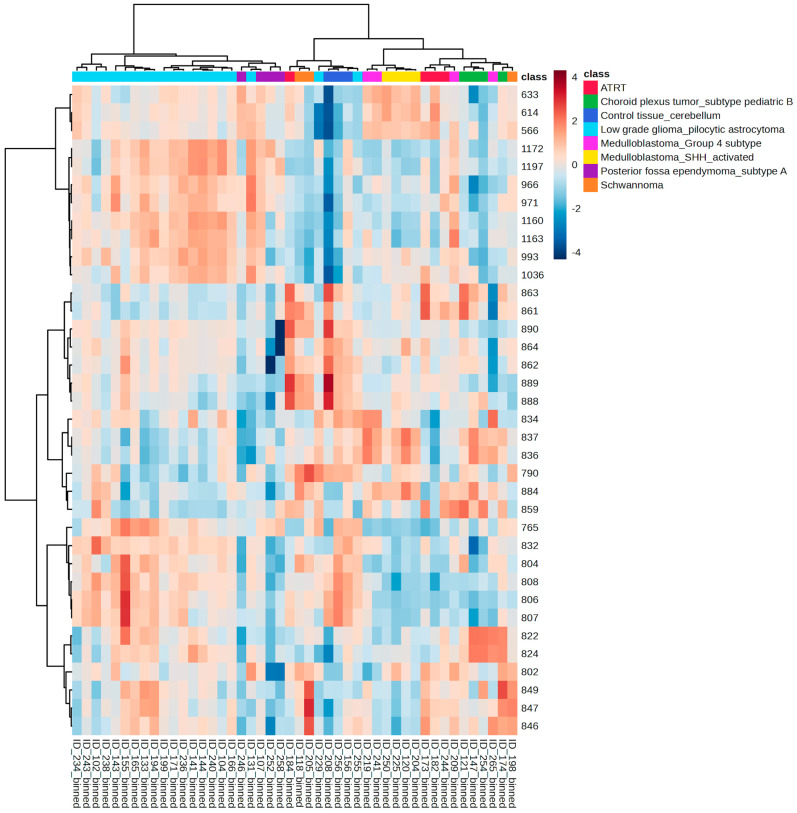
Hierarchical clustering analysis based on the Ward linkage method and Pearson distance depicting the unsupervised grouping of samples based on their lipid profile, according to their respective methylation classes. The color bar represents the data values across the samples, where blue means lower ion abundance and red means higher ion abundance.

An unsupervised hierarchical clustering analysis based on the lipid profile allowed the identification of a cluster of glioma tumors at the left side of the panel, with subsequent division between pilocytic astrocytoma and ependymoma subtype A. Medulloblastoma tumors clustered together, with a further split into Group 4 and SHH-activated subgroups. Choroid plexus tumors subtype B and atypical teratoid rhabdoid tumors (ATRT) also clustered accordingly, at the right side of the panel. Control tissue clustered at the center of the panel, displaying a higher abundance of the ions of *m/z* 888 and 834, typically found in white and gray matter of normal brain parenchyma, respectively (Figure 5).

Despite the clear clustering trend, the number of molecular classes is very large (currently more than 80 classes) and, therefore, our cohort has few representatives in each class. Nevertheless, our results suggest that larger cohorts can potentially reveal distinct chemical profiles for each molecular class of pediatric brain tumors.

## 4. Discussion

In this study, we used DESI-MSI to investigate molecular differences between tumor-transformed tissue and non-tumor tissue in a large cohort of diverse pediatric brain tumors. The 2D chemical images obtained by DESI-MSI were highly correlated to the histological findings present in the brain tumor biopsies. This enabled us to build correlation models between the chemical composition and the tissue histology of the samples. Such models can be trained, allowing a lipid profile to be used as a classifier of samples based on the presence of molecules such as fatty acids or glycerophospholipids [41]. Lasso modeling of our data indicated that it is possible to distinguish tumor-transformed tissue from non-tumor tissue by using DESI-MSI with an accuracy of 96.2% in the training set and 94.3% in the validation set (AUC = 0.96).

Precise brain tumor resection is a fundamental prognostic factor in pediatric neuro-oncology, in association with histology. In general, resection aims at safe gross total resection, and intraoperative imaging tools and intraoperative pathology play a substantial role during the surgical procedure [75]. Friable, fat-rich tissues in the brain are difficult to cut and produce good quality cryosections, which may affect the interpretation of the slides. Regarding suspected intracranial lesions, the agreement between intraoperative frozen section pathology diagnoses and final section diagnoses is variable. Considering a single dataset from a single-center study, agreement was seen in 90.3% of cases (n = 558 biopsies). However, agreement is lower in low-grade gliomas (82.5%) than in high-grade gliomas (93.2%) and even lower in re-do operations (81.5%) [13]. Data presented here suggest that incorporation of DESI-MSI analysis as an assistive intraoperative tool in prospective clinical trials for pediatric brain tumors could be useful to the pathologist’s decision-making process during the intraoperative analysis of frozen sections and, ultimately, may improve the accuracy of surgical handling.

Eberlin et al. (2012) used DESI-MSI to classify gliomas based on their lipid composition and discriminated between oligodendroglioma, astrocytoma, and oligoastrocytoma, demonstrating DESI’s potential in providing diagnosis and information on tumor margins [57]. Santagata et al. (2014) highlighted the role of metabolites like 2-hydroxyglutarate (2-HG) in distinguishing between glioma subtypes and its ability to identify isocitrate dehydrogenase 1-mutant (IDH) tumors, by mapping tissue sections of surgically resected gliomas with DESI-MSI [76]. Cooks and co-workers advanced by testing negative and positive ionization modes of DESI-MS [55] and then opted for the use of negative mode to profile lipids and metabolites from the brain to differentiate gray and white matter, gliomas, meningiomas, and pituitary tumors [52].

Aiming at speeding up the analysis time, DESI-MS was tested for rapid profiling of tissue smears. In this respect, Cooks and co-workers compared the performance of DESI-MSI and DESI-MS rapid analysis of tissue smears for glioma and normal brain tissue and showed the equivalence of the results [54]. They also validated the capability of DESI-MS to differentiate the IDH mutation status of the tumor via detection of 2-HG and to examine the resection cavity walls for residual tumor, estimating tumor cell percentage (TCP) at surgical margins with 93% sensitivity and 83% specificity [53]. The same authors performed DESI-MS in tissue smears to identify IDH mutation status, glioma diagnosis, and estimation of tumor cell infiltration intraoperatively in a large human glioma cohort [56]. Other AIMS techniques, such as PIRL-MS, were also demonstrated as valuable for brain tumor diagnosis. Woolman et al. (2024), while inspecting pediatric brain tumor samples, differentiated between medulloblastoma and pilocytic astrocytoma, as well as two ependymoma molecular subtypes with high sensitivity (94%) and specificity (99%), indicating a significant advancement in cancer classification precision [36].

One of the major gaps in research addressed by our study is the lack of robust evidence to support the incorporation of novel approaches, like DESI-MSI, in molecular margin assessment of pediatric brain tumors. Given the relative lower incidence when compared to adult cancer, pediatric brain series are usually (i) small in numbers [77], (ii) biased towards medulloblastoma, one of the most common pediatric CNS tumors [35,36], or (iii) based on cell line xenografts [78], weakening the power of findings and hindering advancements in the field, since biomarkers relevant for adult cancers and prediction models generated for these patients cannot be automatically transposed to pediatric cancer patients [79].

Our results also demonstrate that prospective and retrospective samples can be jointly analyzed by DESI-MSI, with no apparent bias. In the present study, we included a retrospective clinical case who had disease recurrence at the time of collection of the prospective cohort, allowing us to analyze his tumor at diagnosis and at recurrence, 17 years apart (Supplementary Figure S9). This finding not only opens the possibility of designing specific studies to assess the chemical profile of tumors at diagnosis and at recurrence, aiming at uncovering drug resistance mechanisms and new therapeutic targets, but allows studies to be designed using fresh and banked tissue in the same batch analysis, strengthening the power of analysis.

Although chemical identification of the lipid species is not necessary for a correlation to histopathology data, the identification of these compounds is an important step to gain a deeper understanding of the biochemical processes associated with malignant transformation. Tentative lipid attribution here was based on nominal mass, but previous findings support our data. The ion of *m/z* 794, strongly associated with the tissue changes accompanying tumor transformation, was identified as the chloride adduct of phosphatidylcholine (PC 34:1). Increase in the abundance of *m/z* 794 (PC 34:1) was also reported in other cancers, notably in glioma samples when compared to normal brain tissue from adult patients and prostate cancer [55,80]. An increase in the abundance of *m/z* 885 was also noted in tumor tissue from our series (Figure 1B,F). Our tentative identification of this ion as phosphatidylinositol (PI 38:4) is in accordance with previous DESI studies, which also described higher abundance of *m/z* 885 (PI 38:4) in glioma samples from adult patients, as well in the glioblastoma U87 xenograft model [52,57,81]. The notable increase in the abundance of *m/z* 794 and *m/z* 885 in pediatric brain tissue modified by tumor growth was accompanied by a suppression of *m/z* 834, (PS 40:6) (Table 1, Figure 1C,F), characteristic of normal parenchyma gray matter [52,55]. As per the evidence collected in our study, we observed an overlap between the relevant ions identified by Lasso modeling in our cohort and those previously identified in adult brain tumor patients [52,55]. Such correlation supports the hypothesis of a common panel of biomarkers to be used in neuro-oncology surgery facilities for brain tissue assignment. However, to fully address whether a single model could be employed for both adult and pediatric brain biopsy categorization, a joint clinical study should be conducted.

Brain tumors harboring mutations in genes IDH1 or IDH2 can be identified by DESI-MS due to the accumulation of the oncometabolite 2-hydroxyglutarate (2-HG) [56,76]. Likewise, our evidence suggests that pediatric brain tumors expressing known clinically relevant phenotype biomarkers present a distinct lipid profile. Tumors immunopositive for CD34, BCL2, P53, EGFR, IGF1R, and VEGF showed a typical clusterization pattern based on their lipid profile when compared to negative tumors. In order to precisely identify those lipids differentially abundant between the categories, a large number of tumors should be analyzed. This information is not only relevant for patient prognosis and therapy assessment, but might add up information to the discrimination models based in DESI-MSI data.

In 2021, the WHO adopted the methylation profile as the gold standard method for the molecular classification of CNS tumors [2]. After profiling 128 tumors from our cohort, we found that the lipid profile of tumors showed a clear tendency to cluster according to their methylation-based molecular profile, though the study of a larger number of tumors belonging to each of the different molecular classes will be necessary to definitively establish this association. To our knowledge, this is the first time that DESI-MSI has revealed that the lipid profile of CNS tumors is related to its methylation-based molecular classification. This finding is of utmost clinical relevance and supports further advance in multi-omics studies combining methylome and metabolome datasets to provide real-time intraoperative molecular information from tumors.

In summary, our results strongly suggest that tumor-promoted histology changes can be distinguished from non-tumor tissue in pediatric brain biopsies by using DESI-MSI, irrespective of the tumor diagnosis. Our findings not only made a valuable contribution to the concept of molecular margin assessment, supporting the incorporation of DESI-MSI as an intraoperative assistive tool in prospective clinical trials for management of pediatric neuro-oncology, but positioned these tumors into the vanguard of technical development together with adult glioma, oral squamous cell carcinoma, pancreas, and gastric cancer, for which the value of DESI-MSI as an intraoperative assistive tool was already proved [47,48,57,71].

## Figures and Tables

**Table 1:** DESI-MSI/Lasso prediction results for 25,674 pixels in the training set and 10,734 pixels in the validation set, in comparison with histopathological analyses.

Training set performance	Pathology	DESI-MSI prediction		Agreement
		Normal	non-Normal	
	Normal	1285	146	90%
	non-Normal	830	23,413	97%
		Overall Accuracy: 96.3%		
Validation set performance	Pathology	DESI-MSI prediction		Agreement
		Normal	non-Normal	
	Normal	364	124	75%
	non-Normal	493	9753	95%
		Overall Accuracy: 94.2%		

**Table 2:** Molecular identification of features selected by Lasso as significant contributors to the model for discrimination between tumor-transformed and normal pediatric brain tissue, with attributed statistical weights.

	Feature (Nominal *m/z*)	MS/MS Matched Fragments (Delta)	Putative Lipid Assignment	Proposed Formula	Exact *m/z*	Lasso Weight
Features associated with tumor-promoted changes	794		PC(34:1)	C_42_H_82_ClNO_8_P	794.5472	0.118
		744.55 (0.48)				
		506.33 (0.91)				
		480.31 (0.11)				
	722	436.25 (0.02)	PE(35:5) or PE(P-36:4)	C_40_H_70_NO_8_P or C_41_H_74_NO_7_P	722.4766	0.077
		303.23 (0.07)				
		259.24 (0.23)				
	760	673.48 (0.11)	PS(34:1)	C_40_H_76_NO_10_P	760.5129	0.073
		281.25 (0.04)				
		255.23 (0.09)				
Features associated with histologically normal brain parenchyma	834	747.50 (0.17)	PS(40:6)	C_46_H_78_NO_10_P	834.5285	−0.282
		419.26 (0.26)				
		283.26 (0.04)				
	835		PI(34:1)	C_43_H_81_O_13_P	835.5342	−0.236
		579.29 (0.07)				
		553.28 (0.12)				
		417.24 (0.22)				
		391.23 (0.13)				
		281.25 (0.01)				
		255.23 (0.06)				
		241.01 (0.03)				

**Table 3:** Distribution of tumor samples analyzed according to their methylation-based molecular classification.

Methylation Classes	N. Patients
Low grade glioma, pilocytic astrocytoma subtype	20
Atypical teratoid rhabdoid tumor	4
Choroid plexus tumor, subtype pediatric B	4
Posterior fossa ependymoma, subtype A	4
Medulloblastoma, Group 4 subtype	4
Medulloblastoma, SHH subtype	4
Control tissue, cerebellum	3
Schwannoma	3
**Total**	**46**

## Data Availability

The data presented in this study are available upon request from the corresponding author, given the ethics committee approval to execute the study does not apply to making raw data and related clinical and demographic information publicly available.

## Supplementary Materials

The following supporting information can be downloaded at: https://www.mdpi.com/article/10.3390/biomedicines12112593/s1, Supplementary Figure S1A. DESI-MSI analysis of two tumor samples diagnosed as medulloblastoma. The left panels show the frozen section before the DESI-MSI analysis. The right panels show the 2D chemical image generated from the DESI-MSI analysis, evidencing the distribution of ion 885. The comparison between the prospective and retrospective sample shows that there is no significant difference in the ionization and resolution of the image generated when the two forms of collection are compared. Supplementary Figure S1B. Unsupervised analysis (Principal Component Analysis, PCA) of the data obtained from the prospective and retrospective samples. Supplementary Figure S1C. The nonlinear dimensionality reduction analysis using the t-SNE algorithm shows that it is not possible to distinguish the two groups of samples in relation to the type of recruitment; Supplementary Figure S2. Representative results of ionization failures. In the two left panels, it is observed a lack of correlation between the chemical image generated and the frozen section analyzed. On the right panels, it is possible to observe the chemical spectra obtained demonstrating the relative abundance of ions between *m*/*z* 180 and *m*/*z* 1200. As a result of inadequate ionization, nonspecific ions can significantly interfere with the interpretation of results, evidenced by the background noise observed in the spectra; Supplementary Figure S3. Hierarchical clustering analysis based on complete clustering method and Euclidean distance representing the 25 main variables responsible for grouping the samples into the respective groups: tumors with CD34-positive vessels below and above the median; Supplementary Figure S4. Hierarchical clustering analysis based on complete-linkage clustering and Euclidean distance representing the 25 main variables responsible for grouping the samples into the respective groups: positive and negative tumors for BCL2 immunoexpression; Supplementary Figure S5. Hierarchical clustering analysis based on Ward clustering method and Pearson distance representing 25 variables responsible for grouping the samples into the respective groups: positive and negative tumors for p53 immunoexpression; Supplementary Figure S6. Hierarchical clustering analysis based on Ward clustering method and Euclidean distance representing the main 20 variables responsible for grouping the samples into the respective groups: positive and negative tumors for EGFR immunoexpression; Supplementary Figure S7. Hierarchical clustering analysis representing the main 25 variables responsible for grouping the samples into the respective groups: positive and negative tumors for IGF1R immunoexpression; Supplementary Figure S8. Hierarchical clustering analysis based on complete-linkage clustering and Euclidean distance representing the 25 main variables responsible for grouping the samples into the respective groups: positive and negative tumors for VEGF immunoexpression; Supplementary Figure S9. DESI-MSI analysis of tumor samples from the same patient, at the time of initial diagnosis (year 2003) and at tumor recurrence (year 2020). The left panels depict the chemical images generated from DESI-MSI analysis of frozen sections, evidencing the distribution of the *m*/*z* 788 ion in the tissue. The right panels show the chemical spectra of representative areas of the analyzed tissues, evidencing a high similarity between the chemical profile of the samples, with slight alterations in the relative abundances of the ions. *X* axis: m/z range 180–1200. *Y* axis: relative abundance of ions. The pathology diagnosis confirmed tumor recurrence; Supplementary Table S1: Histopathology diagnosis of patients included in the study; Supplementary Table S2: List of primary antibodies used in the immunohistochemical studies; Supplementary Table S3: List of 119 differentially abundant ions found between normal and tumor tissues; Supplementary Table S4: List of 104 differentially abundant ions found between low- and high-grade tumors.
