# Supplementary material for: Tumor-Promoted Changes in Pediatric Brain Histology Can Be Distinguished from Normal Parenchyma by Desorption Electrospray Ionization Mass Spectrometry Imaging
Source: Biomedicines. 2024 Nov 13;12(11):2593. doi: 10.3390/biomedicines12112593 (PMC11592165; doi:10.3390/biomedicines12112593)
Supplement: Supplementary file 1 [file biomedicines-12-02593-s001.zip › biomedicines-3192548-supplementary.pdf]

Supplementary Data

Supplementary Figure 1A DESI-MSI analysis of two tumor samples diagnosed as medulloblastoma. The left panels show the frozen section before the DESI-MSI analysis. The right panels show the 2D chemical image generated from the DESI-MSI analysis, evidencing the distribution of ion 885. The comparison between the prospective and retrospective sample shows that there is no significant difference in the ionization and resolution of the image generated when the two forms of collection are compared. **Supplementary Figure 1B** Unsupervised analysis (Principal Component Analysis, PCA) of the data obtained from the prospective and retrospective samples. **Supplementary Figure 1C** The nonlinear dimensionality reduction analysis using the t-SNE algorithm shows that it is not possible to distinguish the two groups of samples in relation to the type of recruitment

Supplementary Figure 1

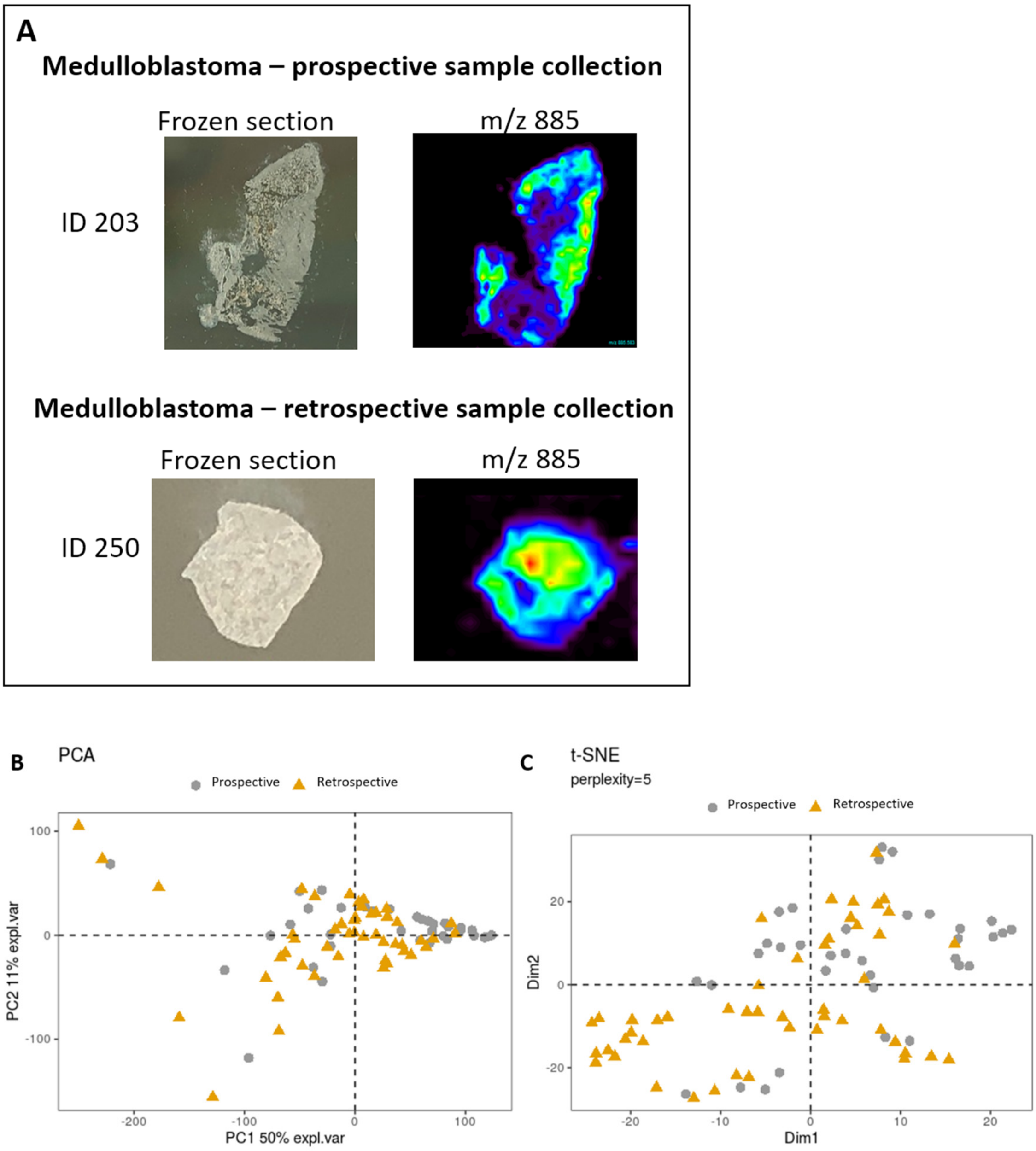

Supplementary Figure 2 Representative results of ionization failures. In the two left panels, it is observed a lack of correlation between the chemical image generated and the frozen section analyzed. On the right panels, it is possible to observe the chemical spectra obtained demonstrating the relative abundance of ions between m/z 180 and m/z 1200. As a result of inadequate ionization, nonspecific ions can significantly interfere with the interpretation of results, evidenced by the background noise observed in the spectra.

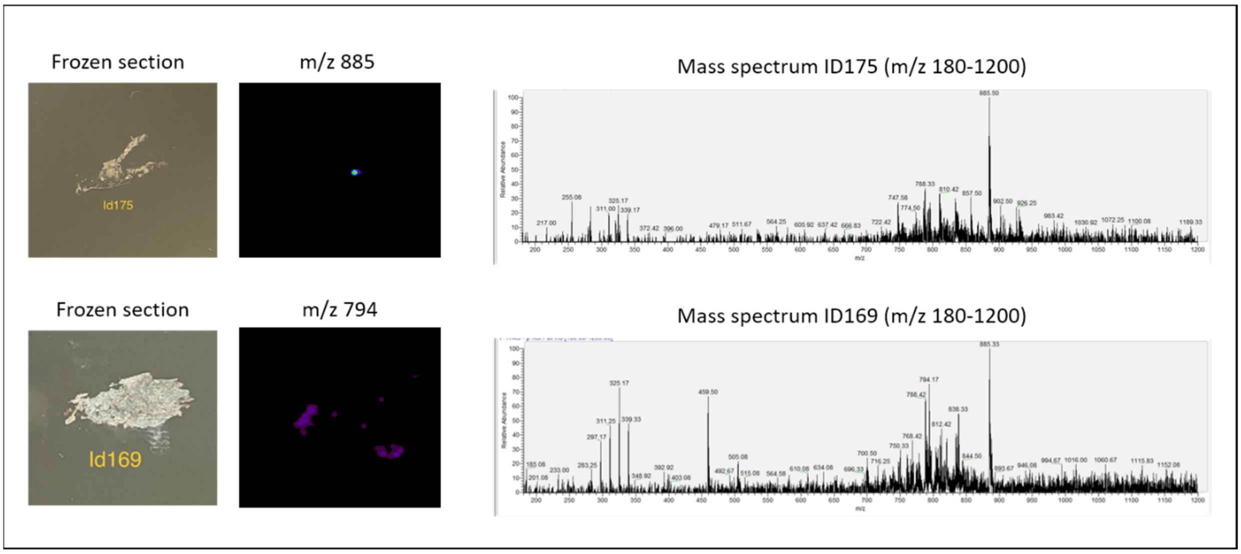

Supplementary Figure 3 Hierarchical clustering analysis based on complete clustering method and Euclidean distance representing the 25 main variables responsible for grouping the samples into the respective groups: tumors with CD34-positive vessels below and above the median. The color bar represents the data values across the samples, where blue means lower ion abundance and red means higher ion abundance

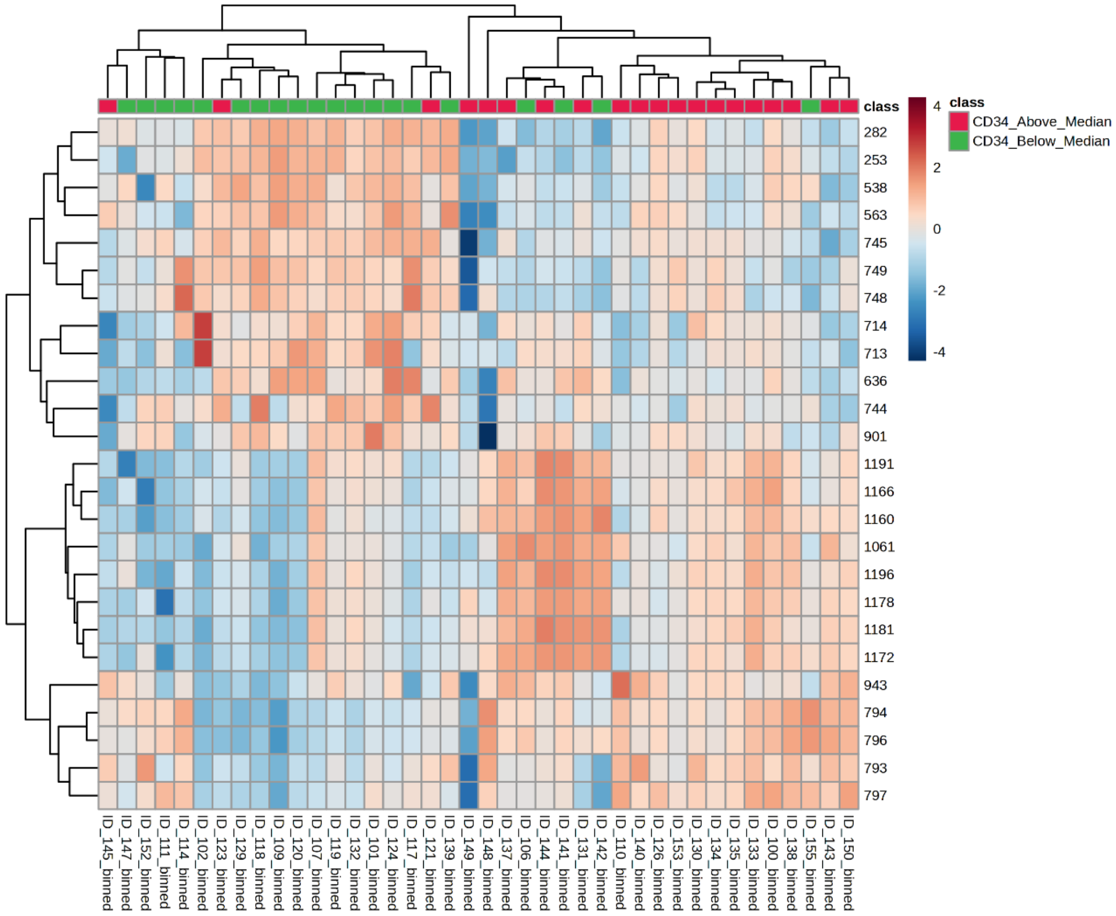

Supplementary Figure 4 Hierarchical clustering analysis based on complete-linkage clustering and Euclidean distance representing the 25 main variables responsible for grouping the samples into the respective groups: positive and negative tumors for BCL2 immunoexpression. The color bar represents the data values across the samples, where blue means lower ion abundance and red means higher ion abundance

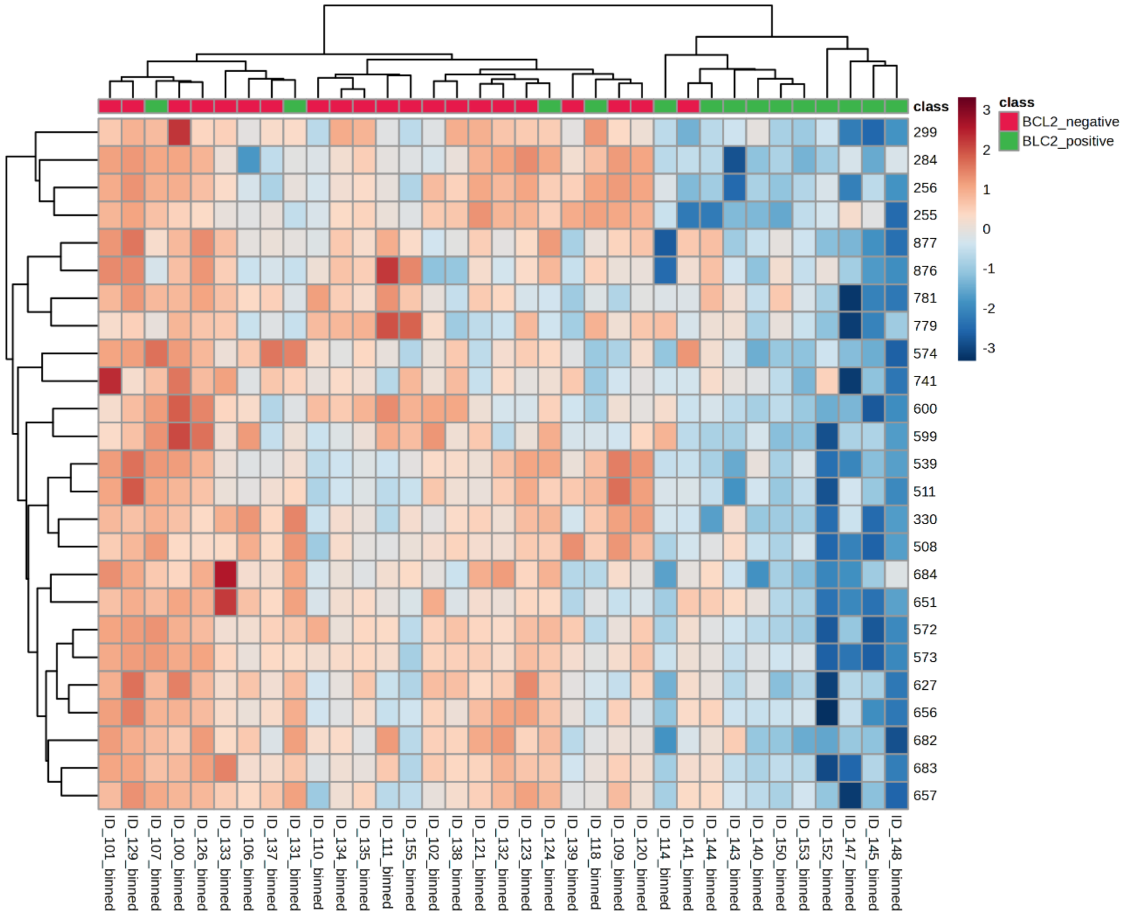

Supplementary Figure 5 Hierarchical clustering analysis based on Ward clustering method and Pearson distance representing 25 variables responsible for grouping the samples into the respective groups: positive and negative tumors for p53 immunoexpression. The color bar represents the data values across the samples, where blue means lower ion abundance and red means higher ion abundance

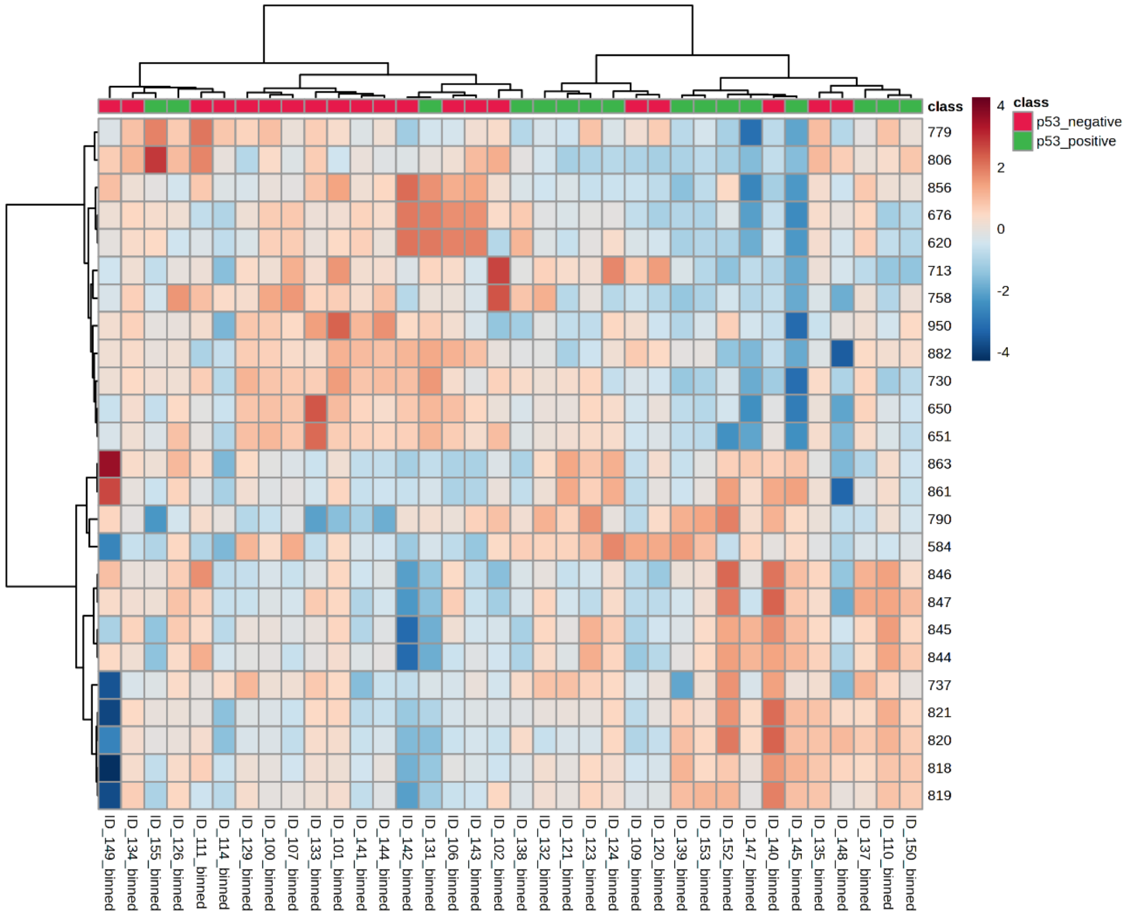

Supplementary Figure 6 Hierarchical clustering analysis based on Ward clustering method and Euclidean distance representing the main 20 variables responsible for grouping the samples into the respective groups: positive and negative tumors for EGFR immunoexpression. The color bar represents the data values across the samples, where blue means lower ion abundance and red means higher ion abundance

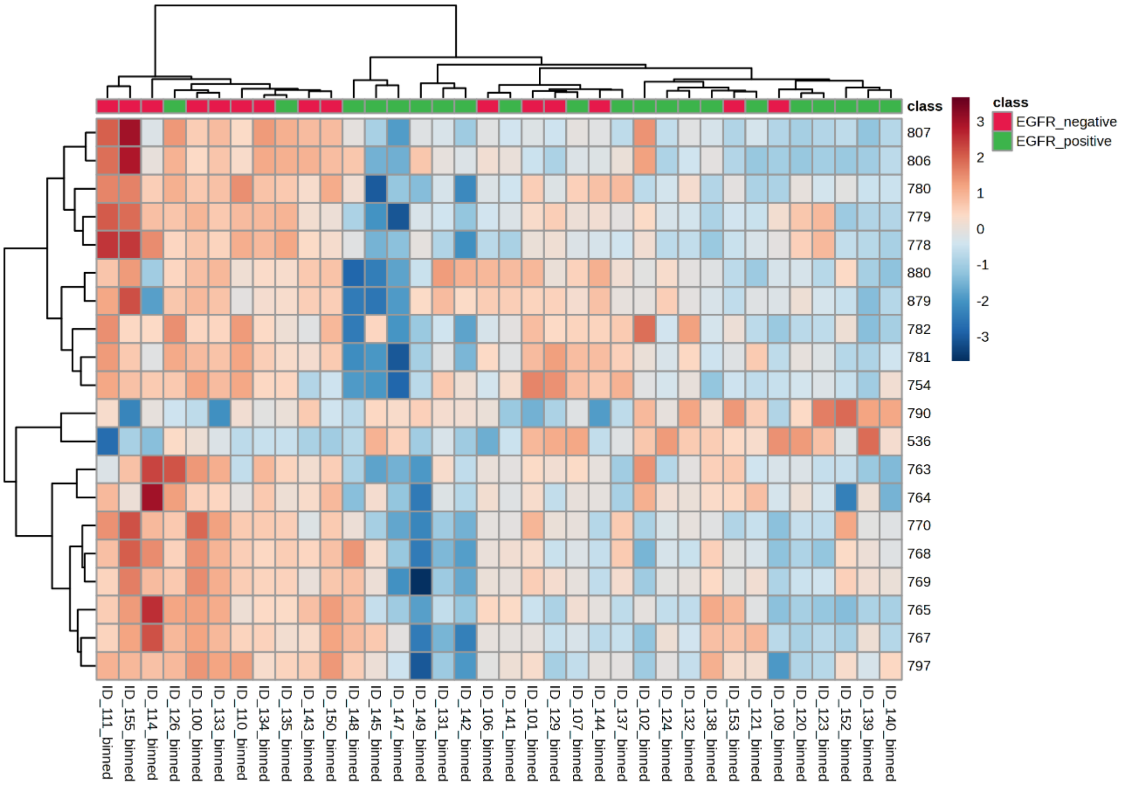

Supplementary Figure 7 Hierarchical clustering analysis representing the main 25 variables responsible for grouping the samples into the respective groups: positive and negative tumors for IGF1R immunoexpression. The color bar represents the data values across the samples, where blue means lower ion abundance and red means higher ion abundance

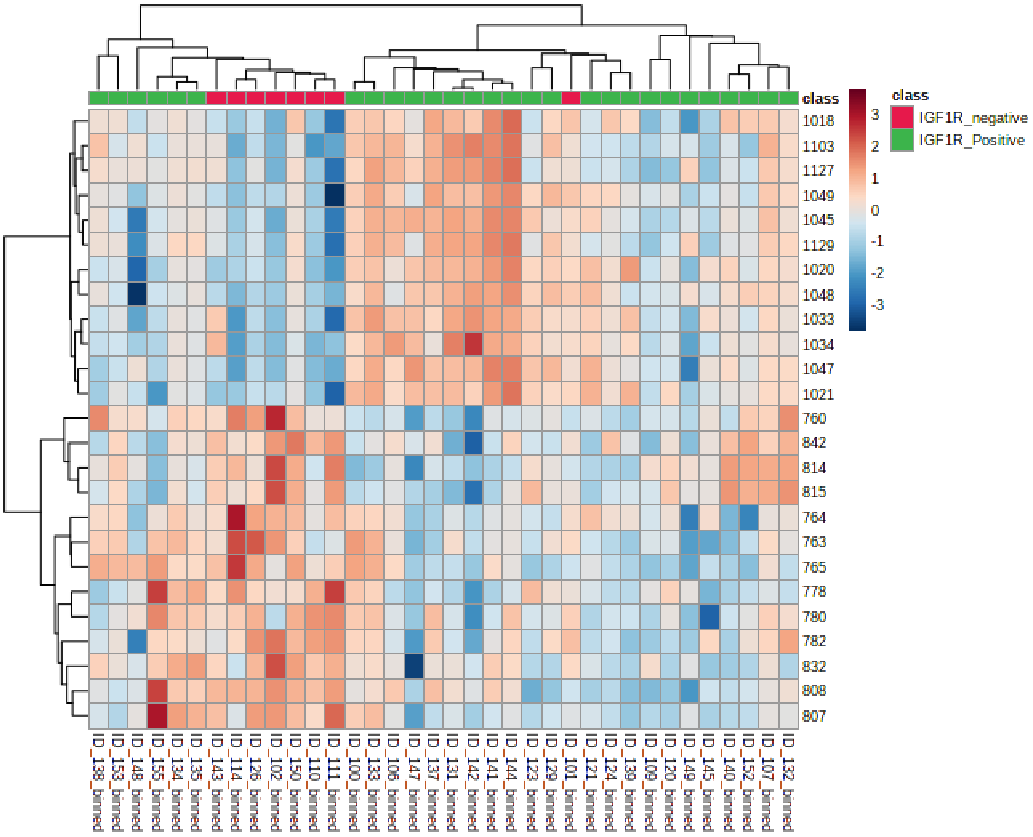

Supplementary Figure 8 Hierarchical clustering analysis based on complete-linkage clustering and Euclidean distance representing the 25 main variables responsible for grouping the samples into the respective groups: positive and negative tumors for VEGF immunoexpression. The color bar represents the data values across the samples, where blue means lower ion abundance and red means higher ion abundance

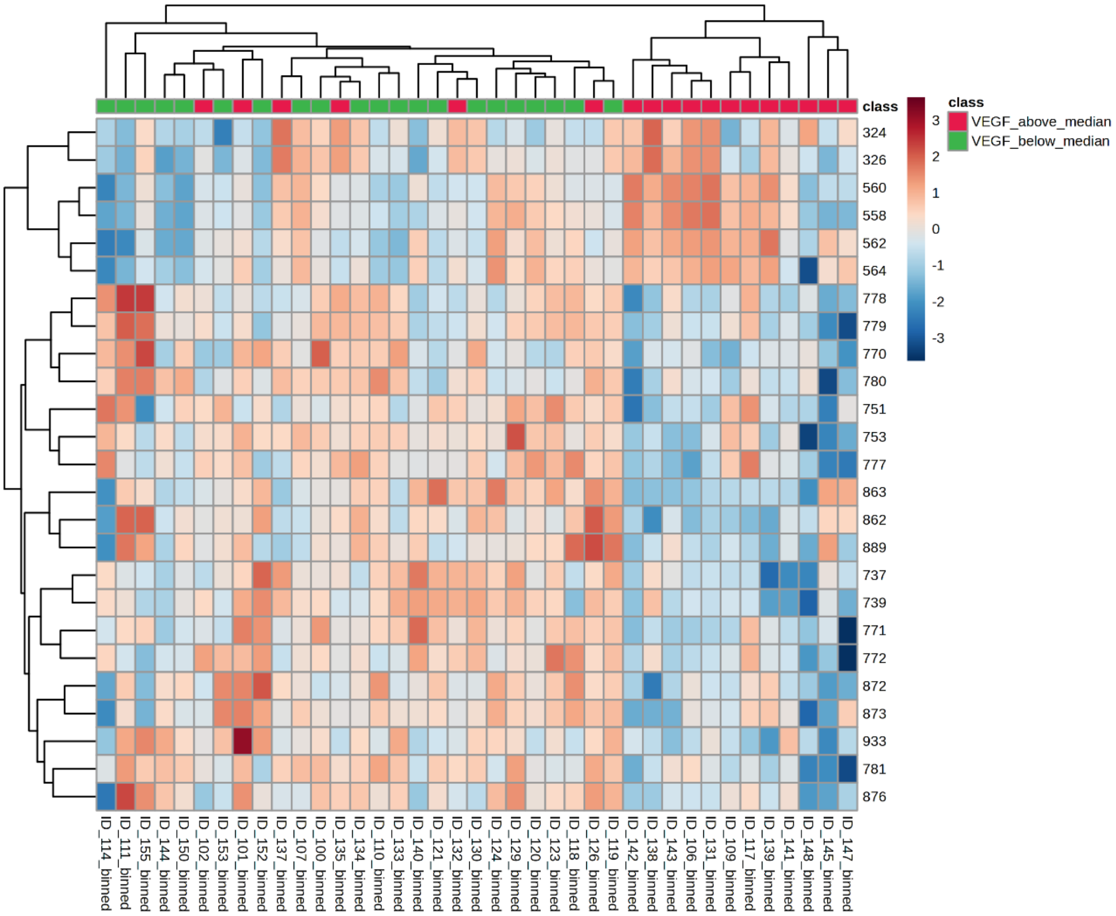

Supplementary Figure 9 DESI-MSI analysis of tumor samples from the same patient, at the time of initial diagnosis (year 2003) and at tumor recurrence (year 2020). The left panels depict the chemical images generated from DESI-MSI analysis of frozen sections, evidencing the distribution of the *m/z* 788 ion in the tissue. The right panels show the chemical spectra of representative areas of the analyzed tissues, evidencing a high similarity between the chemical profile of the samples, with slight alterations in the relative abundances of the ions. X axis: *m/z* range 180-1200. Y axis: relative abundance of ions. The pathology diagnosis confirmed tumor recurrence

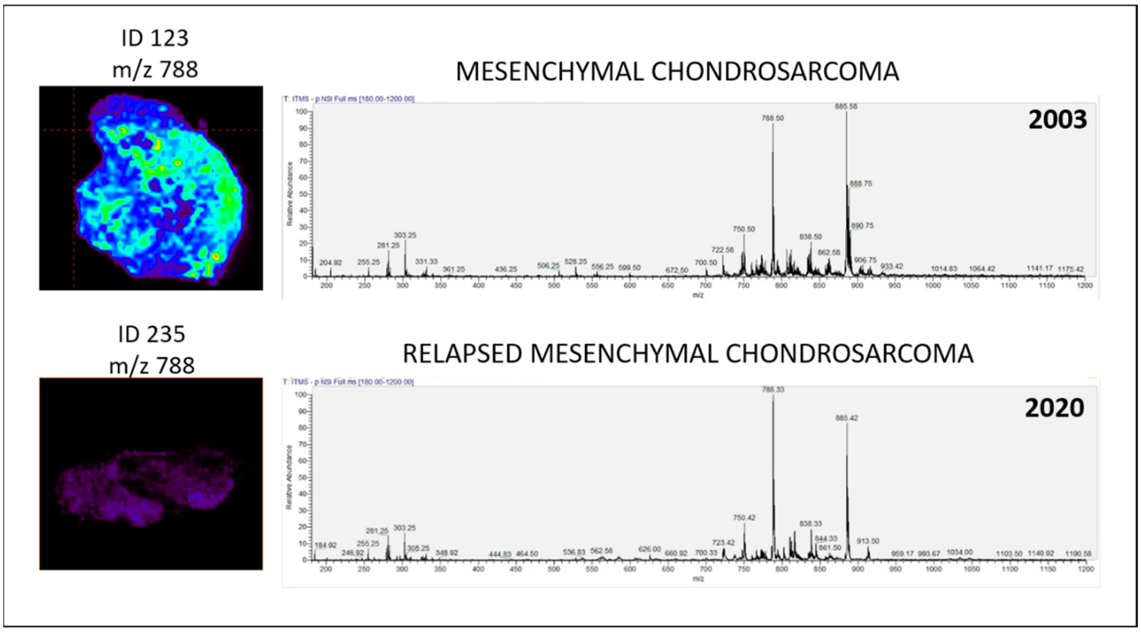

Supplementary Table 1: Histopathology diagnosis of patients included in the study.

	N. Patients
Primary CNS tumor	129
Metastatic	3
Total	132
**Histology**	**N. patients**
ASTROCYTOMA*	37
MEDULLOBLASTOMA	21
EPENDYMOMA	11
CRANIOPHARYONGIOMA	7
GANGLIOGLIOMA	7
CHOROID PLEXUS CARCINOMA	6
DNET	6
SCHWANNOMA	3
Other**	34
Total	132

* This group includes both low-grade and high-grade astrocytomas.

** Tumors that were diagnosed in two or less patients were not detailed separately, it were included in the "other" category.

Supplementary Table 2: List of primary antibodies used in the immunohistochemical studies.

STAINING PURPOSE	ANTIBODY ANTI-	DETAILS	SUPPLIER
**Angiogenesis**	CD34	clone QBEnd/10	Agilent DAKO
	VEGF *	clone VG1	Biogen / Agilent DAKO
**Cell proliferation**	KI-67	clone SP6	Agilent DAKO
**Apoptosis**	Bcl-2	clone E17	Agilent DAKO
	Bax	clone 2D2	Biogen
**Oncogene**	ALK	SP8	Agilent DAKO
**Tumor supressor**	P53	DO7	Agilent DAKO
**Growth factor**	EGFR *	Clone EP22	Biogen
	PDGFR-alpha*	Policlonal	Biogen / Analítica (ABCAM)
	IGF2*	Policlonal	Biogen / Analítica (ABCAM)
	IGF1R*	Policlonal	Biogen / Sigma
**Cell adhesion**	Beta-catenin	Beta-catenin-1	Agilent DAKO
	E-cadherin	NCH-38	Agilent DAKO

*VEGF: *Vascular Endothelial Growth Factor*, EGFR: *Epidermal Growth Factor Receptor*, PDGFR-alpha: *Platelet-derived growth factor receptor*, *alpha polypeptide*, IGF2: *Insulin-like growth factor* 2, IGF1R: *Insulin-like growth factor 1 receptor*.

Supplementary Table 3: List of 119 differentially abundant ions found between normal and tumor tissues.

	FC	log2(FC)	p.ajusted	-LOG10(p)
**917**	4.7238	2.2399	3.3248e-07	6.4782
**918**	4.4968	2.1689	3.3248e-07	6.4782
**907**	5.6393	2.4955	8.1611e-07	6.0883
**834**	4.7777	2.2563	8.1611e-07	6.0883
**891**	4.3275	2.1135	8.1611e-07	6.0883
**835**	4.117	2.0416	8.1611e-07	6.0883
**919**	3.8221	1.9344	8.1611e-07	6.0883
**892**	3.5793	1.8397	8.1611e-07	6.0883
**909**	2.6647	1.414	1.1828e-06	5.9271
**906**	5.419	2.438	1.2743e-06	5.8947
**916**	4.4783	2.163	1.2743e-06	5.8947
**902**	4.0079	2.0028	1.2743e-06	5.8947
**893**	3.1033	1.6338	1.4943e-06	5.8256
**890**	3.8488	1.9444	8.2145e-06	5.0854
**920**	3.5264	1.8182	1.0061e-05	4.9974
**932**	2.6524	1.4073	1.0061e-05	4.9974
**823**	0.404	-1.3076	1.2727e-05	4.8953
**908**	3.2613	1.7054	1.4438e-05	4.8405
**800**	0.37898	-1.3998	1.8294e-05	4.7377
**905**	5.0311	2.3309	2.3176e-05	4.635
**904**	4.6448	2.2156	2.4088e-05	4.6182
**903**	4.1732	2.0612	2.4088e-05	4.6182
**842**	0.44306	-1.1744	2.4088e-05	4.6182
**771**	0.39107	-1.3545	2.6602e-05	4.5751
**741**	0.38348	-1.3828	3.8906e-05	4.41
**740**	0.39589	-1.3368	4.7675e-05	4.3217
**934**	2.4429	1.2886	6.4425e-05	4.1909
**833**	2.4369	1.285	6.4425e-05	4.1909
**877**	2.2784	1.188	6.4425e-05	4.1909
**921**	2.9584	1.5648	8.4545e-05	4.0729
**801**	0.41949	-1.2533	8.4765e-05	4.0718
**824**	0.46317	-1.1104	9.3978e-05	4.027
**889**	3.7451	1.905	0.00012402	3.9065
**943**	0.31812	-1.6524	0.00014874	3.8276
**802**	0.46846	-1.094	0.00014874	3.8276
**933**	2.1339	1.0935	0.00014987	3.8243
**796**	0.36597	-1.4502	0.00015603	3.8068
**822**	0.44799	-1.1585	0.00020491	3.6884
**769**	0.48178	-1.0536	0.0002088	3.6803
**793**	0.48145	-1.0545	0.00031651	3.4996
**770**	0.47805	-1.0648	0.00032966	3.4819
**768**	0.4142	-1.2716	0.0004169	3.38

**795**	0.42282	-1.2419	0.00042134	3.3754
**794**	0.37339	-1.4212	0.00045764	3.3395
**742**	0.49786	-1.0062	0.00046292	3.3345
**1001**	0.39646	-1.3348	0.00067758	3.169
**830**	0.4747	-1.0749	0.00075771	3.1205
**722**	0.24726	-2.0159	0.0009791	3.0092
**684**	0.31917	-1.6476	0.0011437	2.9417
**828**	0.45325	-1.1416	0.0019575	2.7083
**829**	0.48496	-1.0441	0.0025639	2.5911
**184**	0.095344	-3.3907	0.0030785	2.5117
**944**	0.44611	-1.1645	0.0036553	2.4371
**1174**	0.38278	-1.3854	0.0039031	2.4086
**999**	0.48119	-1.0553	0.0040602	2.3915
**1000**	0.47221	-1.0825	0.0042234	2.3743
**324**	0.14119	-2.8243	0.0042399	2.3726
**888**	2.512	1.3288	0.0044119	2.3554
**630**	3.4324	1.7792	0.0044748	2.3492
**1176**	0.36692	-1.4465	0.004539	2.343
**945**	0.47128	-1.0853	0.0059659	2.2243
**758**	0.48153	-1.0543	0.006052	2.2181
**1002**	0.49154	-1.0246	0.0072131	2.1419
**1047**	0.49294	-1.0205	0.008632	2.0639
**1184**	0.41694	-1.2621	0.008678	2.0616
**970**	0.39313	-1.3469	0.0088574	2.0527
**698**	0.42239	-1.2434	0.0088574	2.0527
**984**	0.48291	-1.0502	0.0093451	2.0294
**1060**	0.48801	-1.035	0.0098588	2.0062
**968**	0.40112	-1.3179	0.010877	1.9635
**572**	0.3451	-1.5349	0.011109	1.9543
**1059**	0.46411	-1.1075	0.011109	1.9543
**1179**	0.4211	-1.2478	0.01128	1.9477
**732**	0.40404	-1.3074	0.011541	1.9378
**1178**	0.39411	-1.3433	0.01172	1.9311
**631**	3.1387	1.6502	0.012274	1.911
**339**	0.33395	-1.5823	0.012779	1.8935
**607**	2.6691	1.4163	0.012779	1.8935
**1061**	0.46897	-1.0924	0.01298	1.8867
**1196**	0.40767	-1.2945	0.0133	1.8762
**311**	0.28632	-1.8043	0.014066	1.8518
**1085**	0.38756	-1.3675	0.014066	1.8518
**598**	2.5126	1.3292	0.014195	1.8479
**1197**	0.39947	-1.3238	0.01646	1.7836
**1175**	0.40248	-1.313	0.01646	1.7836
**325**	0.28484	-1.8118	0.018193	1.7401
**567**	2.329	1.2197	0.018193	1.7401
**600**	3.0792	1.6226	0.019942	1.7002

**1198**	0.42684	-1.2282	0.021228	1.6731
**972**	0.49207	-1.0231	0.0219	1.6596
**1067**	0.4856	-1.0422	0.022243	1.6528
**734**	0.41479	-1.2696	0.022822	1.6417
**608**	2.1461	1.1017	0.022822	1.6417
**278**	0.35445	-1.4964	0.023658	1.626
**606**	2.6139	1.3862	0.023658	1.626
**1182**	0.44854	-1.1567	0.024659	1.608
**1181**	0.44132	-1.1801	0.025974	1.5855
**1084**	0.44156	-1.1793	0.025974	1.5855
**1187**	0.46195	-1.1142	0.028637	1.5431
**1124**	0.49112	-1.0259	0.028637	1.5431
**1113**	0.468	-1.0954	0.029388	1.5318
**1119**	0.49779	-1.0064	0.029388	1.5318
**1151**	0.45316	-1.1419	0.029842	1.5252
**1150**	0.44478	-1.1688	0.030481	1.516
**1083**	0.4729	-1.0804	0.030665	1.5134
**1173**	0.46065	-1.1183	0.031139	1.5067
**1123**	0.44898	-1.1553	0.033309	1.4774
**185**	0.27213	-1.8776	0.033821	1.4708
**1172**	0.47065	-1.0873	0.034188	1.4661
**340**	0.40963	-1.2876	0.034787	1.4586
**1185**	0.46836	-1.0943	0.03595	1.4443
**1177**	0.44413	-1.1709	0.039798	1.4001
**682**	0.45809	-1.1263	0.040076	1.3971
**1180**	0.47548	-1.0726	0.042852	1.368
**309**	0.28127	-1.83	0.044148	1.3551
**1195**	0.4818	-1.0535	0.044148	1.3551
**765**	0.48329	-1.049	0.045805	1.3391
**1147**	0.4436	-1.1727	0.046487	1.3327
**460**	0.27442	-1.8655	0.047179	1.3263

Supplementary Table 4: List of 104 differentially abundant ions found between low- and high-grade tumors.

	FC	log2(FC)	p.ajusted	-LOG10(p)
**806**	0.3352	-1.5769	0.00087815	3.0564
**282**	2.8242	1.4978	0.00087815	3.0564
**1170**	0.39509	-1.3398	0.00087815	3.0564
**281**	2.2044	1.1404	0.00087815	3.0564
**305**	2.9518	1.5616	0.00091175	3.0401
**1169**	0.36689	-1.4466	0.00091175	3.0401
**1175**	0.38821	-1.3651	0.00091175	3.0401
**1157**	0.3932	-1.3467	0.00091175	3.0401
**1159**	0.40505	-1.3038	0.00091175	3.0401
**1174**	0.41485	-1.2693	0.00091175	3.0401
**1155**	0.42681	-1.2283	0.00091175	3.0401
**329**	3.1241	1.6434	0.0009889	3.0048
**1144**	0.35454	-1.496	0.0009889	3.0048
**837**	2.4017	1.2641	0.0009889	3.0048
**1176**	0.43067	-1.2154	0.0009889	3.0048
**1166**	0.45037	-1.1508	0.0009889	3.0048
**253**	3.5582	1.8311	0.0010929	2.9614
**509**	2.043	1.0307	0.001165	2.9337
**1143**	0.34869	-1.52	0.0013145	2.8812
**1199**	0.36731	-1.4449	0.0013145	2.8812
**1085**	0.37684	-1.408	0.0013145	2.8812
**1198**	0.41467	-1.27	0.0013157	2.8808
**1115**	0.42385	-1.2384	0.0013157	2.8808
**1114**	0.46892	-1.0926	0.0013157	2.8808
**307**	4.4184	2.1435	0.0013178	2.8802
**309**	3.6188	1.8555	0.0013178	2.8802
**1147**	0.38572	-1.3744	0.0013178	2.8802
**970**	0.39221	-1.3503	0.0013178	2.8802
**1150**	0.41142	-1.2813	0.0013178	2.8802
**1173**	0.41897	-1.2551	0.0013178	2.8802
**1160**	0.43123	-1.2135	0.0013178	2.8802
**1178**	0.43307	-1.2073	0.0013178	2.8802
**1179**	0.43892	-1.188	0.0013178	2.8802
**1183**	0.44002	-1.1844	0.0013178	2.8802
**1099**	0.44135	-1.18	0.0013178	2.8802
**1152**	0.44347	-1.1731	0.0013178	2.8802
**1100**	0.44735	-1.1605	0.0013178	2.8802
**1167**	0.45168	-1.1466	0.0013178	2.8802
**1172**	0.45375	-1.14	0.0013178	2.8802
**1128**	0.48891	-1.0324	0.0013178	2.8802
**1096**	0.45581	-1.1335	0.0013859	2.8583
**807**	0.41089	-1.2832	0.0014072	2.8516

**1139**	0.47464	-1.0751	0.0014293	2.8449
**1142**	0.38732	-1.3684	0.0014521	2.838
**1101**	0.46651	-1.1	0.0014758	2.831
**1177**	0.41424	-1.2715	0.0015529	2.8088
**1156**	0.43088	-1.2147	0.0015529	2.8088
**1102**	0.44593	-1.1651	0.0015529	2.8088
**1182**	0.43117	-1.2137	0.001611	2.7929
**1153**	0.44262	-1.1759	0.0016413	2.7848
**1148**	0.36006	-1.4737	0.0016468	2.7834
**1123**	0.44566	-1.166	0.0016468	2.7834
**1185**	0.45597	-1.133	0.0016788	2.775
**331**	2.7763	1.4732	0.0017305	2.7618
**1171**	0.39839	-1.3277	0.0017305	2.7618
**1158**	0.43112	-1.2138	0.0017305	2.7618
**1089**	0.4318	-1.2116	0.0017305	2.7618
**1084**	0.43695	-1.1944	0.0017305	2.7618
**1188**	0.44119	-1.1805	0.0017305	2.7618
**1194**	0.45578	-1.1336	0.0017305	2.7618
**1184**	0.4662	-1.101	0.0017305	2.7618
**1191**	0.47395	-1.0772	0.0017305	2.7618
**280**	2.0835	1.059	0.0017305	2.7618
**1146**	0.35202	-1.5063	0.0017598	2.7545
**1193**	0.4722	-1.0825	0.0017981	2.7452
**1186**	0.44241	-1.1765	0.0018152	2.7411
**1116**	0.49272	-1.0212	0.0018152	2.7411
**1145**	0.36457	-1.4557	0.0018965	2.7221
**1086**	0.38547	-1.3753	0.0018965	2.7221
**1151**	0.44734	-1.1605	0.0018965	2.7221
**1125**	0.46279	-1.1116	0.0018965	2.7221
**1121**	0.46798	-1.0955	0.0018965	2.7221
**1097**	0.47238	-1.082	0.0018965	2.7221
**1168**	0.47329	-1.0792	0.0018965	2.7221
**1083**	0.4823	-1.052	0.0018965	2.7221
**1126**	0.4951	-1.0142	0.0018965	2.7221
**1180**	0.43369	-1.2053	0.0019852	2.7022
**765**	0.46611	-1.1012	0.0019852	2.7022
**804**	0.49864	-1.0039	0.0021269	2.6723
**1122**	0.45186	-1.146	0.0021423	2.6691
**1113**	0.47305	-1.0799	0.0021423	2.6691
**1118**	0.48564	-1.042	0.0021423	2.6691
**883**	2.0521	1.0371	0.0021423	2.6691
**1154**	0.4599	-1.1206	0.0021727	2.663
**1181**	0.46891	-1.0926	0.0021727	2.663
**1149**	0.40469	-1.3051	0.0022366	2.6504
**1090**	0.48402	-1.0469	0.0022366	2.6504
**1192**	0.49865	-1.0039	0.0022366	2.6504

**1087**	0.38707	-1.3693	0.0022904	2.6401
**1095**	0.45227	-1.1447	0.0025032	2.6015
**1141**	0.43262	-1.2088	0.0025408	2.595
**1073**	0.49939	-1.0018	0.0025408	2.595
**1140**	0.47137	-1.0851	0.0026417	2.5781
**1117**	0.49373	-1.0182	0.0029295	2.5332
**1195**	0.48694	-1.0382	0.0030227	2.5196
**836**	2.0286	1.0205	0.0034051	2.4679
**1088**	0.38435	-1.3795	0.003571	2.4472
**1187**	0.46654	-1.0999	0.0038348	2.4163
**1190**	0.47398	-1.0771	0.0038348	2.4163
**1197**	0.47917	-1.0614	0.0047447	2.3238
**1132**	0.4948	-1.0151	0.0063024	2.2005
**1189**	0.47862	-1.063	0.0065151	2.1861
**184**	0.12862	-2.9588	0.0073119	2.136
**968**	0.42442	-1.2364	0.0096652	2.0148
